# KSHV induces immunoglobulin rearrangements in mature B lymphocytes

**DOI:** 10.1371/journal.ppat.1006967

**Published:** 2018-04-16

**Authors:** Jennifer Totonchy, Jessica M. Osborn, Amy Chadburn, Ramina Nabiee, Lissenya Argueta, Geoffrey Mikita, Ethel Cesarman

**Affiliations:** 1 Pathology and Laboratory Medicine, Weill Cornell Medicine, New York, NY, United States of Amercia; 2 Biomedical and Pharmaceutical Sciences, Chapman University School of Pharmacy, Irvine, CA, United States of Amercia; University of California Berkeley, UNITED STATES

## Abstract

Kaposi sarcoma herpesvirus (KSHV/HHV-8) is a B cell tropic human pathogen, which is present *in vivo* in monotypic immunoglobulin λ (Igλ) light chain but polyclonal B cells. In the current study, we use cell sorting to infect specific B cell lineages from human tonsil specimens in order to examine the immunophenotypic alterations associated with KSHV infection. We describe IL-6 dependent maturation of naïve B lymphocytes in response to KSHV infection and determine that the Igλ monotypic bias of KSHV infection *in vivo* is due to viral induction of BCR revision. Infection of immunoglobulin κ (Igκ) naïve B cells induces expression of Igλ and isotypic inclusion, with eventual loss of Igκ. We show that this phenotypic shift occurs via re-induction of Rag-mediated V(D)J recombination. These data explain the selective presence of KSHV in Igλ B cells *in vivo* and provide the first evidence that a human pathogen can manipulate the molecular mechanisms responsible for immunoglobulin diversity.

## Author summary

Kaposi sarcoma herpesvirus (KSHV) infection of human B cells is poorly understood. KSHV infection in humans is heavily biased towards B cells with a specific subtype of antibody molecule (lambda light chain rather than kappa light chain). This has been a conundrum in the field for years because there is no known physiological distinction between B cells with different light chains that might provide a mechanism for this bias. Here, we develop a novel system for infecting B cells from human tonsil with KSHV and tracking how the virus alters the cells over time. Using this system, we demonstrate a number of KSHV-driven alterations in B cells, including the fact that KSHV infection of kappa light chain positive B cells drives them to become lambda light chain positive by re-inducing recombination events that are normally restricted to B cell development in the bone marrow. We believe that this study is the first demonstration that a virus can alter immunoglobulin specificity via direct infection of B cells.

## Introduction

Kaposi sarcoma herpesvirus (KSHV), also called human herpesvirus 8 (HHV-8) is the most recently discovered human herpesvirus, and infection with this virus is linked to the development of KSHV-associated malignancy including Kaposi sarcoma, primary effusion lymphoma (PEL) and multicentric Castleman disease (MCD), particularly in the absence of adequate immune surveillance (e.g. HIV disease). [1–4]. Although the association of KSHV infection with pathological lymphoproliferation is well established, very little is known about the early stages of KSHV infection in B lymphocytes and how the virus drives pathology in this niche. Moreover, our understanding of the pathogenesis of MCD is specifically hampered by the lack of an experimental model system. As a human herpesvirus with highly restricted tropism, KSHV does not lend itself to animal models, and the murine homolog of KSHV, MHV68, while extensively used to study the immune response to gamma-herpesvirus infection, lacks many homologs of KSHV proteins, and fails to recapitulate key features of human disease entities, including MCD[5]. Previous studies have performed infection of human B cells with KSHV[6–8] and have observed features consistent with MCD phenotypes during *in vitro* infection[9], but no studies to date have combined high infection efficiency with culture methods allowing detailed longitudinal immunophenotyping of lymphocytes during *de novo* KSHV infection.

During B lymphocyte development, establishment of tolerance in the bone marrow involves the sequential production of immunoglobulin light chain rearrangements by V(D)J-recombination orchestrated by the lymphoid lineage-specific recombinase activating gene (RAG) protein products, Rag1 and Rag2[10]. This phenomenon, termed B cell receptor (BCR) editing begins with rearrangements of the immunoglobulin κ (Igκ) locus, and rearrangements in the immunoglobulin λ (Igλ) locus occur only when Igκ rearrangements fail to produce a functional, non-autoreactive BCR. As such, in the human peripheral B cell repertoire Igκ-expressing B lymphocytes outnumber their Igλ counterparts, and the Igκ/Igλ ratio increases as a function of age[11]. Interestingly, restriction of KSHV infection to Igλ positive B lymphocytes is a long-recognized feature of MCD *in vivo*[12,13] and, although PEL are generally immunoglobulin negative those that express surface immunoglobulins are frequently Igλ [14]. Infection of total tonsil-derived B lymphocytes *in vitro* with KSHV has also shown that infected cells are biased towards Igλ+ cells[9]. This phenomenon is counter-intuitive given the relative abundance of Igκ lymphocytes as infection targets and the fact that the two subsets should be physiologically indistinguishable. As such, the restriction of KSHV infection to Igλ+ lymphocytes remains a conundrum in the field.

In the current study, we developed an *in vitro* model system to determine phenotypic changes associated with KSHV-infection of primary B lymphoyctes from human tonsil tissue. During infection of naïve B lymphocytes, we observe IL-6 dependent acquisition of an IgD+CD27+ immunophenotype, termed variously natural effector[15,16], or marginal zone-like (MZL) B lymphocytes in the literature. Our experiments further reveal that KSHV infection induces *de novo* Igλ expression in Igκ tonsil lymphocytes, resulting in skewed Igλ variable gene usage and significant numbers of isotypically included (Igλ+Igκ+) lymphocytes. We demonstrate that Igλ expression in originally Igκ+ lymphocytes during infection is associated with the re-induction of V(D)J recombination, a phenomenon termed BCR revision. Importantly, these data provide an explanation for restriction of KSHV infection to Igλ+ lymphocytes *in vivo*, and represent the first evidence that a lymphotropic human pathogen can induce BCR rearrangements in mature peripheral lymphocytes.

## Results

**Fig 1:**
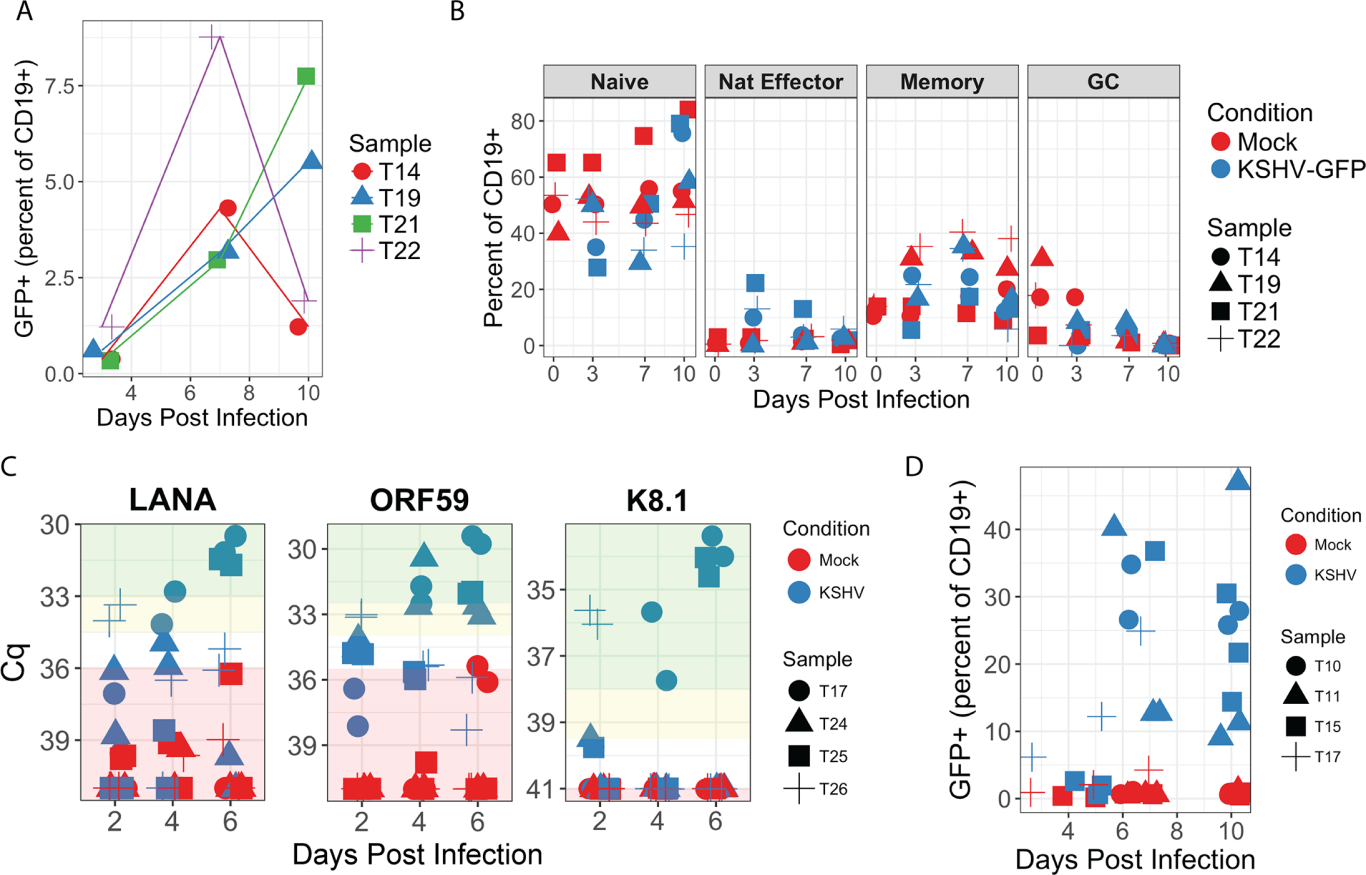
A variety of B lymphocyte lineages from human tonsil are susceptible to infection with BAC16 KSHV. Magnetically sorted total B lymphocytes from four tonsil specimens were infected with KSHV or mock-infected and analyzed by FCM at indicated timepoints for (A) GFP expression and (B) immunophenotypic markers for lineage. In both cases, cells were gated for singlet/viable/CD19+. Memory B cells were further defined as CD38low/IgD-/CD27+, naïve B cells were CD38low/IgD+/CD27-, natural effector (Nat Effector) cells were CD38low/IgD+/CD27+ and germinal center (GC) cells were CD38hi/IgD-. (C) In similar infection experiments with four tonsil specimens, total RNA was extracted at 2, 4 and 6 days post-infection and viral gene transcription was verified in two technical replicates by RT-PCR. Replicate RT negative cDNA reactions for KSHV infected samples at 6 days post-infection were included as a control for DNA contamination and mean NRT Cq values (n = 8) for each target were as follows: 39.44 for LANA, 40.52 for ORF59 and >40 (not detectable) for K8.1. For a 40-cycle reaction, non-amplifying samples were set to Cq = 41 for the purposes of calculation. The lowest Cq value obtained in a mock infected sample was assigned as the limit of detection for each target, and data points that fall below this threshold are denoted with red shading. Yellow shading highlights values between 1.7 and 3.3 cycles lower than the limit of detection and corresponds to 5–10 fold increases in gene expression. Green shading highlights values more than 3.3 cycles lower than the limit of detection and corresponds to gene expression levels greater than 10-fold above the limit of detection. ANOVA analysis of raw Cq values revealed a statistically significant effect of KSHV infection for all target genes when comparing aggregate trends for mock vs KSHV samples over time: LANA p = 0.0006; K8.1 p = 0.02, ORF59 p<0.0001. Student’s T test revealed no significant difference between Cq values for Mock and NRT samples for any target gene. (D) Magnetically sorted naïve B lymphocytes from four tonsil specimens were infected with KSHV or mock-infected and analyzed as in (A) for GFP expression.

For this study, we developed a protocol for flow sorting (S1A Fig) and *in vitro* infection of tonsil-derived primary B lymphocytes with BAC16 recombinant KSHV that met two important criteria: (1) stable infection of sufficient cell numbers to provide phenotypic data by flow cytometry (FCM) and (2) maintenance of pre-sort immunophenotypes in mock-infected controls. Concentrated preparations of cell free recombinant BAC16 KSHV virions were able to infect a variety of B lymphocyte lineages in both bulk infection (Fig 1A and 1B) and isolated subsets flow sorted from human tonsil (S1B Fig), as evidenced by GFP reporter expression from the BAC16 KSHV genome. Co-culture with gamma-irradiated feeder cells expressing FcgRII (CDw32) allowed survival for 2–3 weeks in culture and good maintenance of immunophenotypes in many primary B lymphocyte lineages including naïve (CD19+, CD38low, IgD+, CD27-) and memory (CD19+, CD38low, IgD-, CD27+) cells (S1C Fig). When total B lymphocytes were used for infections, the proportion of specific lineages expressing GFP generally paralleled the overall percentage of the same lineage in corresponding mock-infected cultures, thus revealing no significant bias towards infection of a particular lineage (Fig 1B). Moreover, we were able to validate viral gene expression in total B lymphocyte infections by RT-PCR (Fig 1C). In these assays, KSHV transcript levels generally increased over time in the infected cultures, which is consistent with our observation of increased numbers of GFP+ cells over the same timecourse (Fig 1A). LANA expression >5-fold above the limit of detection was observed for 3/4 tonsil samples, 4/4 samples displayed greater than 5-fold increases in ORF59 and greater than 10-fold increases in K8.1 expression were observed in 3/4 tonsil samples. Taken together, these data indicate that tonsil-derived B lymphocytes are susceptible to recombinant KSHV infection *in vitro* and that there is consistent early lytic gene expression and frequent late lytic gene expression associated with these infections, which may indicate a mixture of lytic and latently infected cells in these mixed B lymphocyte cultures.

Having established a robust infection model, we sought to characterize immunophenotypic changes induced by KSHV infection in B lymphocytes. We used naïve B lymphocytes for the remainder of the experiments described herein because they displayed two features ideal for tracking virus-induced phenotypic changes over time. First, naïve cells were highly susceptible to KSHV infection (Fig 1D and S1B Fig) with variable levels of infection based on the virus stock and tonsil sample used, but generally showing infection rates >10% by 7 days post-infection. Second, uninfected cells robustly maintained their pre-sort immunophenotype over time in culture (S1C Fig). Moreover, previous histological studies have shown data consistent with KSHV infection of naïve B lymphocytes in MCD [12], and infection of the related Epstein-Barr virus in humans (EBV) is thought to fist occur in naïve B cells in the oral mucosa [17].

**Fig 2:**
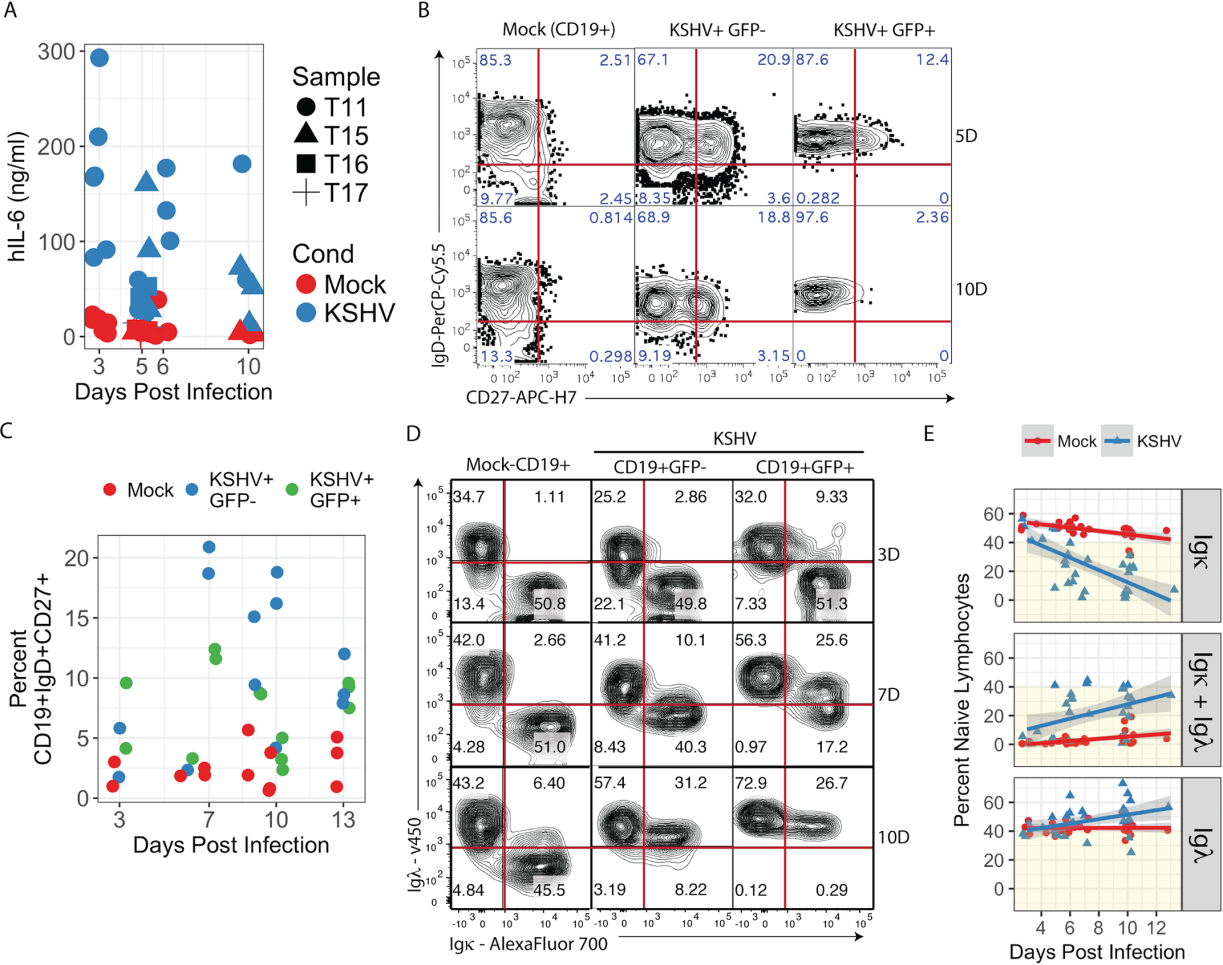
KSHV infection of naïve B lymphocytes recapitulates features of MCD. (A) Concentrations of human IL-6 in culture supernatants were determined in mock and KSHV-infected naïve B lymphocyte cultures at timepoints between 3 and 10 days post-infection by bead-based immunoassay (BD Cytokine Bead Array). Data represent 9 independent experiments with 4 tonsil specimens. p<0.0001 for aggregate data comparing Mock vs. KSHV. p = 0.003 for Mock vs. KSHV at 5 days post-infection and p = 0.004 for Mock vs. KSHV at 6 days post-infection. Primary naïve B lymphocytes were magnetically sorted from total tonsil lymphocytes and infected with KSHV or Mock infected. At timepoints between 3 and 13 days post-infection 2e5 cells were removed from each culture and analyzed by FCM (B) representative plots gated on single, CD19+ population from a representative experiment at 5 and 10 dpi. (C) Aggregate data for 6 independent experiments from 5 tonsil specimens showing the percent of each subset, which expressed both IgD and CD27 at indicated timepoints post-infection. Additional linear mixed model regression on each independent experiment followed by ANOVA (Type II Wald F tests with Kenward-Roger df) analysis revealed a significant effect of KSHV infection (F = 16.5, p = 0.0005) and both GFP negative (p<0.0001) and GFP positive (p = 0.02) populations were significantly different from Mock based on post-hoc Tukey test. **(**D) FCM plots gated on CD19+GFP- or CD19+/GFP+ populations from a representative experiment at 3, 7 and 10 dpi. (E) Aggregate data for 11 experiments from 8 individual tonsil specimens showing the percent of the CD19+ population with each light chain phenotype (Igκ+, Igκ+Igλ+ and Igλ) over the timecourse of infection. Linear regression was performed in R software using least means method and gray shading represents a 95% confidence interval. Additional linear mixed model regression on each independent experiment followed by ANOVA (Type II Wald F tests with Kenward-Roger df) analysis revealed significant effects of KSHV infection for each light chain immunophenotype (for Igκ: F = 147.7, p = 2.3E-15; for Igκ+Igλ: F = 52.4, p = 6.3E-9; for Igλ: F = 16.5, p = 0.0002).

In our KSHV-infected naïve B lymphocyte cultures we observed several reproducible immunophenotypic shifts. There were consistently elevated levels of IL-6 in culture supernatants (Fig 2A), We employed a species-specific cytokine assay in order to ensure that cytokines were being secreted by B cells and not the murine feeder cells. In contrast, levels of IL-2, IL-4, IL-10 and TNFα remained unchanged in the same supernatants. Concurrently, we observed upregulation of CD27 during KSHV infection producing a substantial population of cells with an IgD+CD27+ immunophenotype (Fig 2B & 2C), termed variously natural effector or marginal zone-like (MZL) B lymphocytes in the literature[15,16]. These results are consistent with a previous study demonstrating both IL-6 production and CD27 acquisition in tonsil lymphocytes infected with PEL-derived wild-type KSHV[9]. Interestingly, although statistically significant trends in IgD+CD27+ subsets were observed in both GFP+ and GFP- populations in KSHV-infected cultures compared to mock-infected cultures, the stronger effect occurred in the GFP- (bystander) population (Fig 2B and 2C). Circulating MZL B cells are currently the subject of considerable interest and debate, and are thought to represent a T cell-independent, extrafollicular maturation pathway[18]. Moreover, both IL-6 secretion and MZL B lymphocyte phenotypes are seen in KSHV-infected cells in primary cases of MCD[12,19]. Thus, our *in vitro* infection system recapitulates key features of KSHV infection *in vivo* and MCD pathogenesis, supporting the conclusion that KSHV infection may drive an extrafollicular maturation pathway.

At early times post infection, we observe that KSHV infects both Igκ+ and Igλ+ naïve B cells equally (Fig 2D, top right panel). However, later in infection we see profound shifts in immunoglobulin light chain expression. Specifically, we found a substantial loss of Igκ+Igλ- lymphocytes coincident with the emergence of an isotypically included (Igκ+Igλ+) subset and an increased proportion of Igκ-Igλ+ cells (Fig 2D & 2E). Interestingly, both the GFP+ and GFP- cells in the infected cultures lost Igκ expression, indicating that the phenotype may be influenced by a soluble factor.

**Fig 3:**
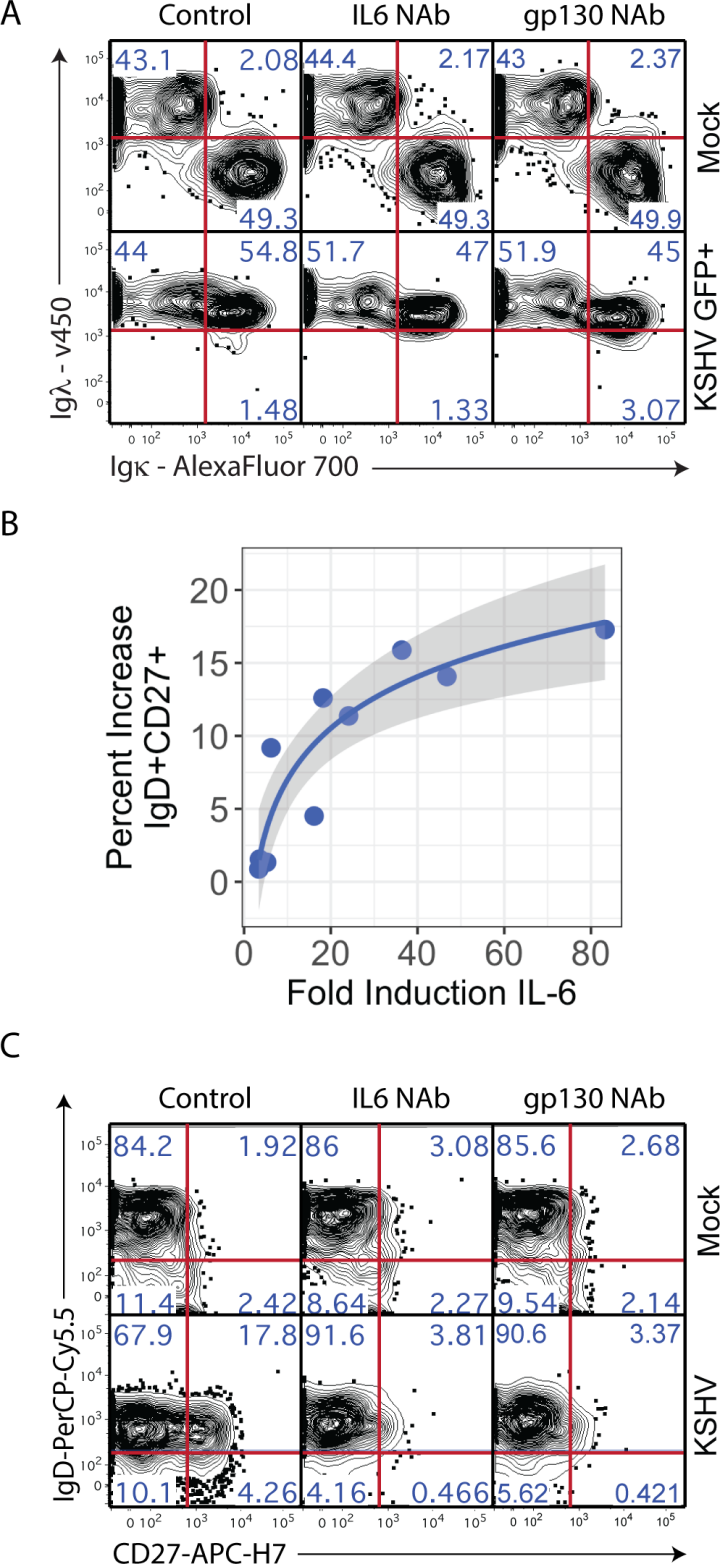
IL-6 signaling does not affect BCR revision, but controls MZL phenotype in KSHV-infected cultures. (A) Naïve B lymphocyte cultures were infected with KSHV or Mock infected and treated with PBS (Control), IL6 neutralizing antibody at 200ng/ml or gp130 neutralizing antibody at 2μg/ml. At 6 days post-infection, cells were analyzed by FCM and subsets were gated as in Fig 2D. Results were verified in two independent experiments with two tonsil specimens. (B) Aggregate data for 10 individual observations from 6 independent experiments correlating the induction of IL6 (KSHV/Mock) from Fig 2A with the MZL (IgD+CD27+) population in the same sample at the same timepoint. Log regression was performed in R software using least means method (*r*^2^ = 0.82, p = 0.0003) and gray shading represents a 95% confidence interval. (C) Mock or KSHV infected naïve B lymphocytes were treated with IL-6 or gp130 antibodies and analyzed as in Fig 1B. Plots shown are from a singlet/viable/CD19+CD38low gating hierarchy in a representative experiment and results were verified in two independent experiments with two tonsil specimens.

Because IL-6 signaling is known to be a critical driver of pathogenesis in MCD[19], we determined whether the immunophenotypic alterations we observe *in vitro* were a result of IL-6 signaling. Neutralization of either hIL-6 or the gp130 receptor had no effect on KSHV-mediated shifts in Ig light chain expression (Fig 3A). However, we observed that the emergence of the MZL immunophenotype (IgD+CD27+) in our infected cultures was highly correlated with the magnitude of IL-6 induction (Fig 3B), and neutralization of hIL-6 or its receptor abrogated the induction MZL phenotypes in KSHV-infected cultures (Fig 3C), indicating that the induction of hIL-6 by KSHV drives B cell maturation. Moreover, cells in which light chains were modified by KSHV (isotypically included and revised) did not display alterations in CD27 expression, suggesting that CD27 acquisition and light chain revision are mutually exclusive phenomena in our experiments.

**Fig 4:**
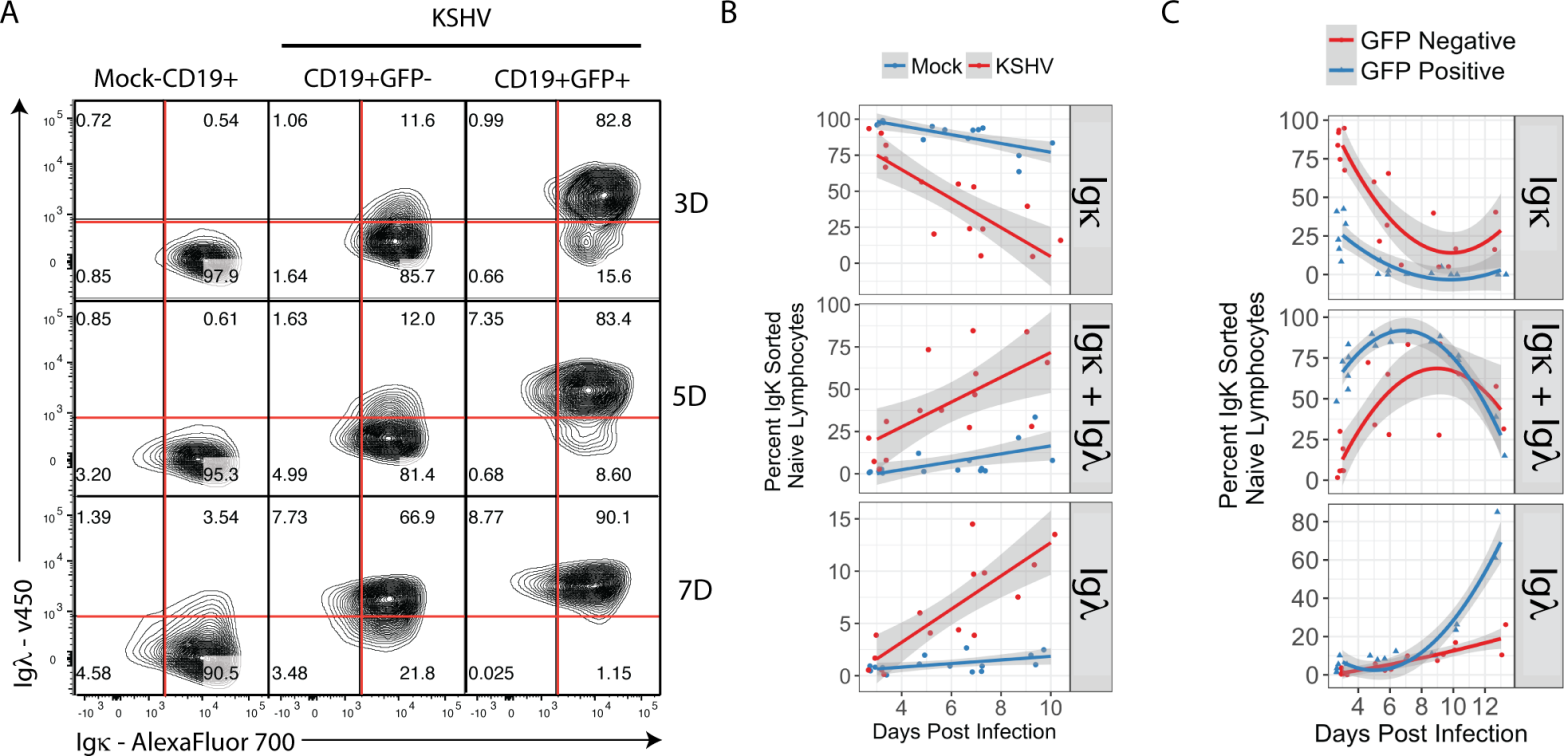
KSHV-infected Igκ+ lymphocytes become Igλ+ via an isotypically included intermediate. Primary naïve B lymphocytes were magnetically sorted from total tonsil lymphocytes, then subsequently flow sorted based on light chain expression and infected with KSHV or mock-infected. At 3, 5 and 7 days post-infection 2e5 cells were removed from each culture and analyzed by FCM for expression of Igκ and Igλ. (A) FCM plots gated as shown in S2A Fig from a representative experiment. (B) Aggregate data for 6 experiments from 6 individual tonsil specimens showing the percent of the single, viable, CD19+ population with each light chain phenotype (Igκ+, Igκ+Igλ+ and Igλ+) based on gates shown in (A) over the time course of infection. Linear regression on the aggregate data was performed in R software using least means method and gray shading represents a 95% confidence interval. Additional linear mixed model regression on each independent experiment followed by ANOVA (Type II Wald F test with Kenward-Roger df) analysis revealed highly significant and dominant effects of KSHV infection for each light chain immunophenotype (for Igκ: F = 58.05, p = 5.8E-8; for Igκ+Igλ: F = 27.95, p = 1.8E-5; for Igλ: F = 35.06, p = 3.5E-6). (C) Aggregate data as in (b) for KSHV-infected samples only separating GFP+ and GFP- events. Biexponential regression was performed in R software using least means method and gray shading represents a 95% confidence interval.

In order to determine whether the loss of Igκ+ lymphocytes in KSHV infected cultures is due to selective toxicity or phenotypic shift we performed experiments in which Igκ+ naïve lymphocytes were purified by flow sorting and subsequently infected with KSHV. The full gating scheme for this analysis is provided in S2A Fig. In these experiments, mock-infected Igκ+ cultures displayed no significant shift in Igκ expression over the experimental time course. In contrast, we observe a rapid and profound shift of KSHV-infected Igκ+ lymphocytes to Igκ+Igλ+, which is followed by the emergence of a small but reproducible Igκ-Igλ+ population at later timepoints (Fig 4A and Fig 4B, bottom panel). This phenotype is robust, and the shift for each light chain population (Igκ+, Igκ+Igλ+ and Igλ+) is statistically significant compared to mock infected samples (Fig 4B). As with our unsorted experiments, we observe that GFP- cells in the KSHV infected culture also undergo a significant shift to Igκ+Igλ+ (Fig 4A, middle panels). Interestingly, although both populations display isotypic inclusion, the kinetics are slower in the GFP- population. This bystander effect could be due to a soluble factor other than IL-6 or direct interactions between infected and uninfected cells. Alternatively, the revised GFP- cells could represent a population of lymphocytes in which KSHV infection was aborted at a stage prior to GFP expression. Similar experiments performed in flow sorted Igλ lymphocytes revealed no alterations in light chain expression (S2B Fig). Moreover, we have identified six lymph node biopsies from four HIV+ patients with AIDS-related lymphadenopathy (ARL), which did not have histological features of MCD but had rare KSHV-positive cells. In these cases, like in MCD, the infected cells were Igλ+ (S3 Fig). Thus, the Igλ bias of KSHV infection *in vivo* is a feature of KSHV infection itself, rather than a phenotype specific to MCD. Taken together, these data support the conclusion that the Igλ-bias observed in KSHV infected cells *in vivo* is a result of immunophenotypic shift after infection rather than a bias towards infection of Igλ+ lymphocytes.

**Fig 5:**
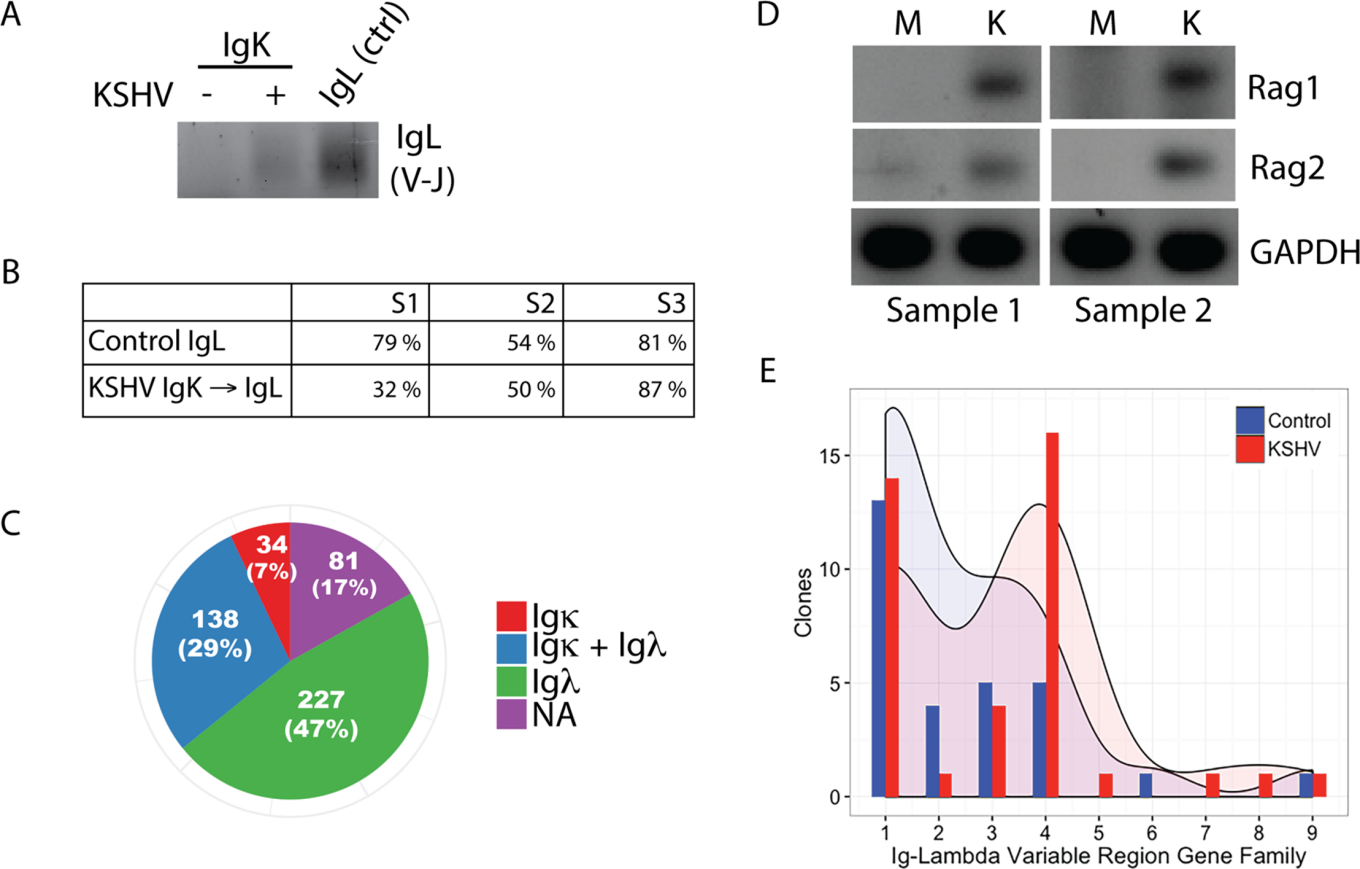
Validation of V(D)J recombination in KSHV infected cultures. (A) Primary naïve B lymphocytes were magnetically sorted from total tonsil lymphocytes, then subsequently flow sorted based on light chain expression and infected with KSHV or mock-infected. At 4 days post-infection total genomic DNA was harvested and BIOMED2 primers were used to amplify functional V-J rearrangements by PCR. (B) Mock Igλ+ or KSHV Igκ+ cultures were flow sorted at 7dpi for Igλ+ (Mock Control) or GFP+Igλ+ (KSHV-modified) and single cells were collected in 96-well PCR plates. RT-PCR was performed from single cell cDNA for Igλ transcripts. Data represents the percent of single cells expressing Igλ transcripts from three individual tonsil donors in three independent experiments. n≥96 for each sample. (C) KSHV Igκ+ cultures were flow sorted at 7dpi and GFP+Igκ+Igλ+ single cells were collected in 96-well PCR plates. RT-PCR was performed from single cell cDNA for both Igλ and Igκ transcripts. Data represents the number of single cells amplifying the indicated light chains or with no amplification (NA), overall n = 480 from three individual tonsil donors in two independent experiments. (D) Primary naïve B lymphocytes were KSHV or mock-infected. Total mRNA was harvested at 4 hours post-infection and RAG1 and RAG2 transcripts were amplified by nested RT-PCR. RT-PCR results were verified in 6 individual tonsil samples. (E) KSHV-modified Igλ transcripts (from Fig 4B, representing single infections from three individual tonsil donors) were sequenced and immunoglobulin lambda variable (IGLV) gene families were determined using IgBLAST (NCBI). Data displays the histogram actual frequency and Gaussian kernel density distribution for each IGLV gene family for Control (Mock Igλ+) and KSHV-modified (Igκ+ at infection, GFP+Igλ+ at sorting) sequences. Kolmogorov-Smirnov test indicates a statistically significant difference between the distributions (p<0.0001).

We next sought to determine whether V(D)J recombination drives the emergence of Igλ expression in KSHV-infected Igκ+ B lymphocytes using the diagnostic BIOMED2 primer set[20]. This is a standard assay to document the presence of V(D)J rearrangements revealing polyclonal and monoclonal B cells. We were able to detect polyclonal V-J genomic rearrangements in the Igλ locus in flow sorted Igλ lymphocytes and in KSHV-infected Igκ+ lymphocytes, but not in mock-infected Igκ+ lymphocytes. This can be appreciated by the intensity of the smear, as expected for a polyclonal B cell population (Fig 5A). We further verified the expression of functionally rearranged Igλ mRNA sequences in KSHV-altered Igκ lymphocytes by nested RT-PCR. For these experiments, we flow sorted Igκ+ lymphocytes, infected them with KSHV and then performed a secondary sort at 7 days post-infection, capturing single cells that were GFP+ and had transitioned to Igκ-Igλ+. cDNA was made from these single cells and Igλ transcripts were amplified by nested RT-PCR. We used the amplification of Igλ transcripts from mock infected Igλ+ controls, which had been sorted for Igλ expression, then mock-infected, cultured and post-sorted in parallel with KSHV samples as a technical control for our ability to detect Igλ expression in single cells for each experiment. Three independent experiments of ≥96 single cells per condition using three distinct tonsil specimens revealed significant numbers of KSHV-modified Igκ lymphocytes expressing Igλ transcripts (Fig 5B). Similarly, we were able to validate the isotypically included (Igκ+Igλ+) population by single cell RT-PCR (Fig 5C). Expression of the lymphocyte-specific recombinases Rag1 and Rag2 is normally restricted to immature B lymphocytes in the bone marrow and the expression of these proteins regulates V(D)J recombination and BCR editing during B lymphocyte development[10]. In order to determine whether KSHV-associated BCR revision was Rag-mediated we performed nested RT-PCR for Rag transcripts and were able to detect both RAG1 and RAG2 mRNA at early time points post-infection (Fig 5D). This result was reproducible in six tonsil specimens at time points <6 hours post-infection. However, due to the very early induction of RAG transcripts which was consistently observed prior to the expression of GFP in our infected cultures, we were unable to determine whether expression of Rag proteins was restricted to KSHV infected cells or was more widespread within the culture. We hypothesized that if V(D)J recombination was being re-induced in these cultures, limiting the repair of Rag-mediated double stranded DNA breaks would result in apoptosis. Indeed, pharmacological inhibition of DNA-PKcs (a critical enzyme in the double stranded break repair pathway used in V(D)J recombination) resulted in selective toxicity in KSHV-infected cultures (S4 Fig). Taken together, these data clearly demonstrate the re-induction of V(D)J recombination activity in mature primary B lymphocytes during *de novo* KSHV infection.

In order to explore the specific nature of the Igλ rearrangements in KSHV-infected B lymphocytes, we used cDNA from flow sorted single cells analyzed in Fig 5B to sequence Igλ variable regions from control (Mock Igλ+) and KSHV-revised (Igκ+ at infection, GFP+ Igκ-Igλ+ at 7dpi) lymphocytes and determined IGLV gene usage using IgBLAST. As expected[21], control Igλ clones were biased towards IGLV gene families 1–3 with few clones utilizing IGLV4-10. In contrast, KSHV-modified Igλ+ cells displayed increased usage of upstream IGLV family genes including a significant number of clones from the VL4 gene family (Fig 5E). Interestingly, despite enriching for functional rearrangements by sorting Igλ-expressing lymphocytes, more of the KSHV-modified Igλ clones we sequenced were non-productive rearrangements (defined as out-of-frame rearrangements or those incorporating a premature stop codon): (6/20) in KSHV-modified samples compared to the Igλ controls (2/17). This increased rate of non-productive rearrangements (although not statistically significant based on the low numbers of antibody clones) is consistent with the high error rate for V(D)J recombination[22], and further supports the conclusion that *de novo* V(D)J recombination is occurring in these cultures. Moreover, the high prevalence of non-productive Igλ rearrangements in KSHV-infected Igκ cells sorted for Igλ expression suggests that mechanisms of allelic exclusion may not be functional in this system.

## Discussion

Our data demonstrating that KSHV induces BCR revision during *de novo* infection of human B lymphocytes is the first evidence that a lymphotropic human pathogen can alter immunoglobulin specificity by direct infection of B lymphocytes. These results could imply that KSHV infection can shape the immune repertoire. MYC-activating translocations involving the immunoglobulin loci present in Burkitt’s lymphoma (BL) were once thought to be a product of EBV-mediated re-induction of V(D)J recombination [23]. While infection of B cells with EBV has been shown to induce expression of RAG1 and RAG2[24], these were not detected in early passage lymphoblastoid cells[25] or most EBV-associated lymphomas[26]. Moreover, there is no apparent bias towards Igλ expression in EBV-associated tumors or EBV-infected cells *in vivo* as is seen with KSHV infection. However, the phenotypic shifts to Igλ we observe during infection *in vitro* explain the longstanding enigma of KSHV restriction to Igλ+ lymphocytes in primary patient specimens. Certainly, the implications of KSHV-mediated alteration of the human immunoglobulin repertoire are potentially far-reaching and deserve further study. KSHV-mediated BCR editing might represent a novel immune-evasion mechanism directed at altering the adaptive immune response to infection. This hypothesis is consistent with clinical evidence that antibody responses to KSHV in infected human populations are inconsistent and highly variable over time[27–29].

For this study, we rely on a new *ex vivo* culture model for primary B lymphocytes in which mouse L cells expressing FCγRII/CDw32 receptor are able to maintain lymphocyte immunophenotypes in isolated B lymphocyte lineages over time. The choice of these feeder cells was largely empirical based on trial and error, and the robust maintenance of naïve B lymphocytes in this system was surprising because naïve cells lack IgG expression and thus should not bind CDw32. This discrepancy certainly deserves further study as it may hint at an uncharacterized quorum sensing mechanism present in lymphoid organs. Although the data presented herein uses primarily naïve B lymphocytes, the target cell type(s) for KSHV infection *in vivo* are still a subject of debate [30]. Indeed, we show that multiple lineages are susceptible to KSHV infection both in bulk (Fig 1B) and sorted cultures (S1B Fig). Certainly, additional studies into the specific biology associated with KSHV infection of a variety of B cell lineages are warranted. Moreover, our use of tonsil lymphocytes derived from routine tonsillectomy may raise questions about the contribution of the baseline inflammatory state and the presence of co-pathogens in inflamed tonsil tissue to KSHV infection. There is epidemiological evidence that KSHV transmission is inefficient[31] and the prevalence of KSHV infection in the developing world is suggestive of a correlation between the presence of co-pathogens and KSHV transmission. As such, we would suggest that lymphocytes from inflamed tonsil specimens represent a relevant model for KSHV transmission.

Based on these results, we propose that KSHV infection represents an interesting experimental method for perturbing human B cell physiology *ex vivo*. A robust *in vitro* infection and culture system, coupled with our ability to manipulate the KSHV genome represents a unique opportunity to use viral subversion of B cell biology to investigate obscure aspects of human immunology. For example, our observation that KSHV-infected cells acquire MZL B cell immunophenotypes dependent upon IL-6 signaling provides novel insight into the development of this enigmatic lineage, and investigations of the KSHV-mediated mechanisms driving the emergence of MZL B cells from naïve progenitors could reveal previously undiscovered pathways regulating extrafollicular B cell maturation in humans. Moreover, BCR revision in human immunology remains highly controversial[32–35], and our current model system using human cells provides a method to study the cell biology underlying this phenomenon.

Interestingly, both isotypic inclusion and BCR revision have been implicated as potential drivers of autoimmune disease[36–38]. This is particularly interesting given that HIV disease[39] and KSHV-associated lymphoproliferative diseases[40–42] frequently co-present with autoimmune manifestations. Although it would be difficult to determine whether KSHV infection plays a direct role in promoting autoimmune disease in humans, study of the B cell-specific pathways targeted by KSHV to affect BCR revision could provide critical insights into the pathogenesis of autoimmune diseases in which BCR revision plays a role.

## Materials and methods

### Reagents and cell lines

IL-6 and gp130 neutralizing antibodies were from R&D systems. NU7441 was from Sigma. FCM antibodies were from BD and are detailed below. CDw32 L cells (CRL-10680) and control L cells (CRL-2648) were obtained from ATCC and were cultured in DMEM supplemented with 10% FBS (Atlanta Biologicals) and 50μg/ml gentamicin. For preparation of feeder cells, L cells were trypsinized and resuspended in 30ml of media in a 50ml conical tube and irradiated with 3000 rad using a gamma source. Irradiated cells were then counted and cryopreserved until needed for experiments.

### Preparation of cell-free recombinant KSHV

iSLK[43] bearing BAC16[44] recombinant KSHV WT (BAC16 producer cells were kindly provided by Ashlee Moses, OHSU) were grown in DMEM supplemented with 10% FBS, 50μg/ml gentamicin, 250μg/ml G418, 1μM Puromycin, and 1.2mg/ml Hygromycin B. Eight confluent T175 flasks were induced for 72 hours with 3mM Sodium Butyrate and 2μM Doxycycline. Culture supernatants were clarified twice by centrifugation at 2000rpm for 5 minutes and 7000rpm for 15 minutes. Virus was pelleted out of clarified supernatants over a 25% sucrose cushion by ultracentrifugation at 22,000 rpm for 90 minutes. Virus pellets were resuspended in a total of 2ml TNE buffer and stored at -80°C.

### Preparation, infection and culture of primary lymphocytes from human tonsil

De-identified primary human tonsil specimens were obtained after routine tonsillectomy from the Weill Cornell/New York Presbyterian Immunopathology Laboratory Biorepository with approval from the Institutional Review Board of Weill Cornell Medical College. Lymphocytes were extracted by dissection and masceration of the tissue in RPMI media. Lymphocyte-containing media was passed through a 70μm filter and pelleted at 400g for 7 minutes. RBC were lysed for 5 minutes in RBC lysing solution (0.15M ammonium chloride, 10mM potassium bicarbonate, 0.1M EDTA). After dilution to 50ml with RPMI, lymphocytes were filtered through a 0.4μm filter, counted and pelleted a second time. Aliquots of 1(10)^8 cells were resuspended in 1ml of freezing media containing 90% FBS and 10% DMSO and cryopreserved. For experiments, lymphocytes were thawed rapidly at 37°C, diluted to 10 ml with 10% RPMI and pelleted at 400g for 5 minutes. Cells were resuspended in 1ml RPMI with 20% FBS and 100μg/ml Primocin (Invivogen) and incubated at 37°C for 4–18 hours. For experiments utilizing magnetic cell sorting, untouched total B cells (Miltenyi Cat# 130-091-151) or naïve B cells (Miltenyi Cat# 130-091-150) were isolated according to manufacturer instructions. For experiments utilizing fluorescence activated cell sorting (FACS), lymphocytes were washed 1x with PBS and resuspended in PreSort Buffer (BD Biosciences) containing antibodies to CD19 (BD Cat# 340720, 16μl/1(10)^6 cells), Immunoglobulin Lambda Light Chain (BD Cat# 561379, 4μl/1(10)^6 cells) and Immunoglobulin Kappa Light Chain (BD Cat# 561319, 4μl/1(10)^6 cells) and incubated on ice for 15 minutes. Cells were washed 2x with PreSort Buffer, resuspended in 2ml PreSort Buffer and sorted on a Custom Order FACSAria Cell Sorter. For infection with KSHV, 1(10)^6 lymphocytes were pelleted into 12x75mm round bottom tubes and resuspended in 400μl serum free RPMI containing TNE (Mock) or KSHV. Doses of virus used for infections were calculated based on per cell infection volume required to achieve 20% infection of HUVEC at 48 hours based on GFP expression, and correspond to 0.1–5 genomes/cell depending upon the specific virus preparation. Cells were centrifuged at 1000rpm in inoculating medium for 30 minutes at 4°C and transferred to 37°C for a further 30 minutes. 100μl FBS (20% of the final culture volume) and Primocin were added and cells with inoculum were transferred to gamma-irradiated mouse L CDw32 feeder cells. At 3 days post-infection, the media was replaced with fresh 20% RPMI+Primocin, removing residual virus inoculum and cells were fed with fresh media every 3 days over the experimental timecourse

### Multi-color flow cytometry for B cell immunophenotyping

At indicated times post-infection a proportion of lymphocyte cultures representing ~200,000 cells were pelleted at 400g for 3 minutes into 96-well round bottom plates. Cells were washed once with PBS and resuspended in 200μl PBS containing (0.4ng/ml) fixable viability stain (BD Cat# 564406) and incubated at room temperature for 10 minutes. Cells were pelleted and resuspended in 100μl cold PBS without calcium and magnesium containing 5% FBS, and 0.1% Sodium Azide (FACS Block) and incubated on ice for 15 minutes after which 100μl cold PBS containing 0.5% FBS and 0.1% Sodium Azide (FACS Wash) was added. Cells were pelleted and resuspended in FACS Wash containing B cell phenotype panel as follows for 15 minutes on ice: (volumes indicated were routinely used for up to 0.5(10)^6 cells and were based on titration of the individual antibodies on primary tonsil lymphocyte specimens) Ig Lambda Light Chain-V450 (2μl), CD19-PE (8μl), CD38-PECy7 (3μl, BD Cat# 560667), IgD-PerCP Cy5.5 (2.5μl, BD Cat# 561315), CD138-APC (4μl, BD Cat# 347207), CD27-APC H7 (2.5μl BD Cat# 560222), Ig Kappa Light Chain-Alexa700 (2μl). After incubation, 100μl FACS Wash was added and pelleted lymphocytes were washed with a further 200μl of FACS Wash prior to being resuspended in 200μl FACS Wash for analysis. Data was acquired on a BD LSR2 Flow Cytometer and analyzed using FlowJo software.

### RT-PCR for viral gene expression

For viral gene expression assays, primary naïve lymphocytes from four independent tonsil specimens were magnetically sorted and infected with KSHV or mock-infected. 1e6 cells were harvested at indicated timepoints and total RNA was extracted using Directzol RNA Miniprep Kit (Zymo Research) according to manufacturer instructions. A second DNase step was performed on 50ng total RNA using Ambion DNA free Kit (Cat #AM1906) and cDNA was synthesized from 50ng total RNA using Thermo High Capacity cDNA synthesis kit. 3μl of cDNA was used for duplicate RT-PCR reactions with Taqman Fast Advanced Mastermix (Cat #: 4444556). Primer and probe (FAM/BGH) sequences were as follows (5’ to 3’): LANA Fwd: GCCTATACCAGGAAGTCCCA, LANA Rev: GAGCCACCGGTAAAGTAGGA, LANA Probe: ACACAAATGCTGGCAGCCCG K8.1 Fwd: TGCTAGTAACCGTGTGCCAT, K8.1 Rev: AGATGGGTCCGTATTTCTGC, K8.1 Probe: TGCGCGTCTCTTCCTCTAGTCGTTG; ORF59 Fwd: TTAAGTAGGAATGCACCCGTT, ORF59 Rev: GGAAGCCGGTGGTAGGAT, ORF59 Probe: CCAGGCTTCTCCTCTGTGGCAA.

### PCR for genomic immunoglobulin rearrangements

Primary naïve lymphocytes were flow sorted based on light chain expression and infected with KSHV, as above. At 4 days post-infection lymphocytes were harvested and genomic DNA was extracted using Wizard SV kit protocol (Promega Cat #A2360) and eluted in 2 x 75-μL water containing 1 μL RNase A. 12.5-ng and 40-ng of extracted genomic DNA was used as PCR template for mock samples and KSHV-infected samples, respectively. 50-μL of Platinum Supermix Hifi (Thermo Cat # 12532) was used, in addition to 0.5-μL of each 10 μM Lambda Forward and Reverse primers (Forward: Vl1/2–5' ATTCTCTGGCTCCAAGTCTGGC 3' and Vl3- 5' GGATCCCTGAGCGATTCTCTGG 3'; Reverse: Jl1/2/3–5' CTAGGACGGTGAGCTTGGTCCC 3'). The PCR program was as follows: 7-minutes at 95°C, 50 cycles of 30-seconds at 95°C, 30-seconds at 64.7°C, and 30-seconds at 72°C, followed by 10-minutes at 72°C [20]. The PCR products from the first amplification were used for a second identical PCR amplification using 2-μL of previous reaction as template. The PCR products from the re-amplification reaction were analyzed on a 2% agarose gel.

### Single cell RT-PCR for immunoglobulin light chains

Single cells were harvested by flow sorting into 96-well PCR plates containing 4μl of RNA lysis buffer (0.5x PBS+10mM DTT+4U SUPERas-In (Thermo Cat #AM2694)). Plates were sealed and stored at -80°C. cDNA was synthesized directly in wells using Thermo High Capacity cDNA synthesis kit (Cat #4368814). PCR primers for amplification of immunoglobulin light chains were from Tiller et. al.[45]. Outer RT-PCR reactions were assembled with Illustra PureTaq Ready-to-go PCR beads (GE Cat #27-9557-02), mixtures of 0.2μl of each 10μM outer primer and 3μl of single cell cDNA in total 25μl reaction volumes. Cycling parameters for outer PCR were 50 cycles of 30-seconds at 95°C, 30-seconds at 60°C for Igλ or 58°C for Igκ, and 55-seconds at 72°C, followed by 5-minutes at 72°C. Nested PCR was assembled with 15μl 2x Phusion Flash Mastermix (Cat # F548L), 0.05μl of each 10μM inner primer mixture and 3μl of outer PCR reaction in 30μl total reaction volume. Primer sequences were adapted from[45,46]. Cycling parameters for nested PCR were 40 cycles of 10-seconds at 95°C, 15-seconds at 60°C for Igλ or 58°C for Igκ, and 10-seconds at 72°C. PCR products were analyzed on 2% agarose gel or purified for Sanger sequencing by isopropanol precipitation.

### RT-PCR for Rag recombinase

Naïve tonsil lymphocytes were magnetically isolated and infected as above. 2(10)^6 mock or KSHV-infected lymphocytes were harvested into 400μl Tri-Reagent at 4 hours post-infection and total RNA was extracted using Directzol RNA Miniprep Kit (Zymo Research) according to manufacturer instructions. A second DNase step was performed on 50ng total RNA using Promega RQ1 DNase kit (Cat #M6101) and cDNA was synthesized from 50ng total RNA using Thermo High Capacity cDNA synthesis kit. Outer RT-PCR reactions were assembled with 30μl of Platinum Hifi Supermix, 0.5μl of each 10μM outer primer (RAG1 Forward: 5’-AAGGAGAGAGCAGAGAACAC-3’, RAG1 Reverse: 5’-GTCCCAACTCAGCCATTGTT-3’, RAG2 Forward: 5’-AGTCAGCCTTCTGCTTGC-3’, RAG2 Reverse: 5’- AGGCAGCTTGGAGTCTGAAA-3’) and 3μl of cDNA. Cycling parameters for outer PCR were 40 cycles of 30-seconds at 95°C, 30-seconds at 53°C, and 45-seconds at 68°C, followed by 5-minutes at 68°C. Nested PCR was assembled with 30μl Platinum Hifi Supermix, 0.5μl of each 10μM inner primer (RAG1 Forward: 5’-TTCTGCCCCAGATGAAATTC -3’, RAG1 Reverse: 5’-CTGGACAAGGCTGATGGTCA-3’, RAG2 Forward: 5’-TCTCTGCAGATGGTAACAGTCAG-3’, RAG2 Reverse: 5’-CTACCTCCCTCCTCTTCGCT-3’) and 3μl of outer PCR reaction. Cycling parameters for nested PCR were 50 cycles of 30-seconds at 95°C, 30-seconds at 64°C for RAG1 and 63°C for RAG2, and 30-seconds at 68°C, followed by 5-minutes at 68°C. For GAPDH the nested PCR reaction was performed with conditions identical to RAG2 using 3μl cDNA. PCR products were visualized on 2% agarose gel

### Bead-based cytokine immunoassay

200μl supernatants were harvested from primary cell cultures at various times post-infection, clarified by centrifugation and stored at -80°C. Supernatants were thawed and subjected to bead-based immunoassay (BD CBA Cat# 561521) according to manufacturer’s instructions. Briefly, standard curve samples were prepared and processed together with experimental samples. Human IL-6 Capture beads (BD Cat# 558276) were incubated with supernatants for 2 hours at room temperature, then detection reagent was added and incubated for another 2 hours at room temperature followed by two washes. Samples were then incubated for 1 hour at room temperature with enhanced sensitivity detection reagent, washed twice and data was acquired by FCM using a BD LSR2 flow cytometer and analyzed using FlowJo software. Two technical replicates were performed for each sample. Standard curves and absolute cytokine values were calculated using R software.

### Immunohistochemistry

Dual immunohistochemistry for KSHV LANA and immunoglobulin light chains was performed as previously described[12].

### Statistics

Data plots and statistical analysis were performed in R software[47] using ggplot2[48] and RColorBrewer[49] packages. Additional statistical analysis was performed on aggregate data using R packages car[50], lme4[51], lmerTest[52]. Specific methods of statistical analysis and resulting values for significance are detailed in the corresponding figure legends.

## Supporting information

S1 Fig Multiple B lymphocyte subsets are susceptible to KSHV infection in vitro. (A) gating scheme for flow sorting of tonsil B lymphocyte lineages: GC (CD19+, CD38hi, IgD-), Naive (CD19+, CD38low, IgD+, CD27-) and Memory (CD19+, CD38low, IgD-, CD27+). (B) KSHV infection over time of flow-sorted B lymphocyte subsets based on GFP fluorescence gated based on a parallel Mock culture at each timepoint. GC were plated on gamma-irradiated CD40L feeder cells, Memory and Naive were plated on gamma-irradiated CDw32 feeder cells. (C) immunophenotypes over time based on the defining criteria for each lineage at sorting (A).
(PDF)

S2 Fig Supplemental material relating to BCR revision. (A) Full gating scheme for flow sorted BCR revision experiments shown in Fig 4 (B) Igλ+ Naive B lymphocytes were sorted, infected with KSHV and analyzed in parallel with Igκ lymphocytes shown in Fig 4.
(PDF)

S3 Fig Igλ restriction is a feature of KSHV infection in both MCD and non-MCD lymph node biopsies. Primary samples with H&E staining at 4x and 20x and corresponding immunohistochemistry for LANA (red) and immunoglobulin light chains (brown) demonstrating that both KSHV-infected lymphocytes (red arrows) in MCD (top) and non-MCD AIDS-related lymphadenopathy (bottom) do not express Igκ (black arrows, left) but are positive for Igλ (black arrows, right).
(PDF)

S4 Fig Inhibition of DNA-PKcs is selectively toxic to KSHV-infected lymphocytes. Naive B lymphocytes were flow sorted based on Igκ-expression and pre-treated with DMSO or 5μM NU7441 for 1 hour. Cells were subsequently infected mock-infected or infected with KSHV in the presence of treatments and plated on irradiated CDw32 feeder cells. At 5 days post-infection cells were harvested and analyzed by FACS for (A) cell viability using an exclusion dye and (B) light chain expression. Singlet-gated viable cells were included in the analysis. For (B) light chain expression for total CD19+ (green) and CD19+, GFP+ (blue) in a representative experiment is shown.
(PDF)
