# Supplementary figures and images for: KSHV induces immunoglobulin rearrangements in mature B lymphocytes
Source: PLoS Pathog. 2018 Apr 16;14(4):e1006967. doi: 10.1371/journal.ppat.1006967 (PMC5919685; doi:10.1371/journal.ppat.1006967)

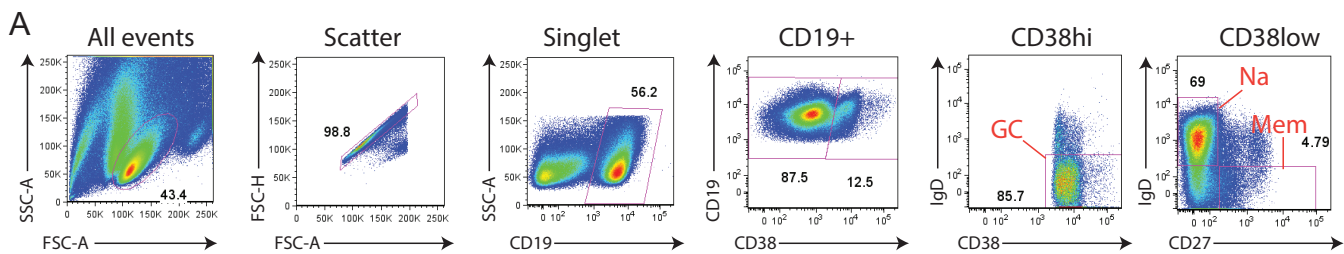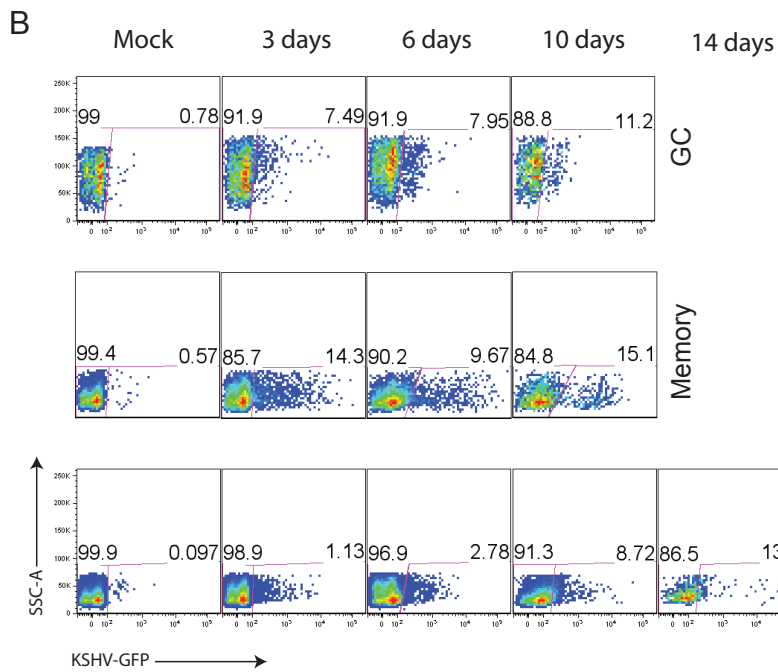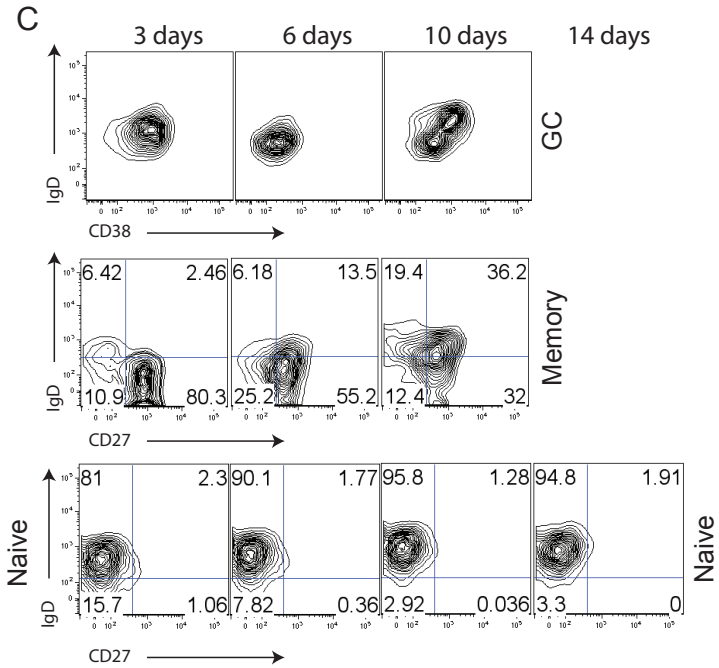

Supplement: S1 Fig — (A) gating scheme for flow sorting of tonsil B lymphocyte lineages: GC (CD19+, CD38hi, IgD-), Naive (CD19+, CD38low, IgD+, CD27-) and Memory (CD19+, CD38low, IgD-, CD27+). (B) KSHV infection over time of flow-sorted B lymphocyte subsets based on GFP fluorescence gated based on a parallel Mock culture at each timepoint. GC were plated on gamma-irradiated CD40L feeder cells, Memory and Naive were plated on gamma-irradiated CDw32 feeder cells. (C) immunophenotypes over time based on the defining criteria for each lineage at sorting (A). (PDF) [file ppat.1006967.s001.pdf]

A

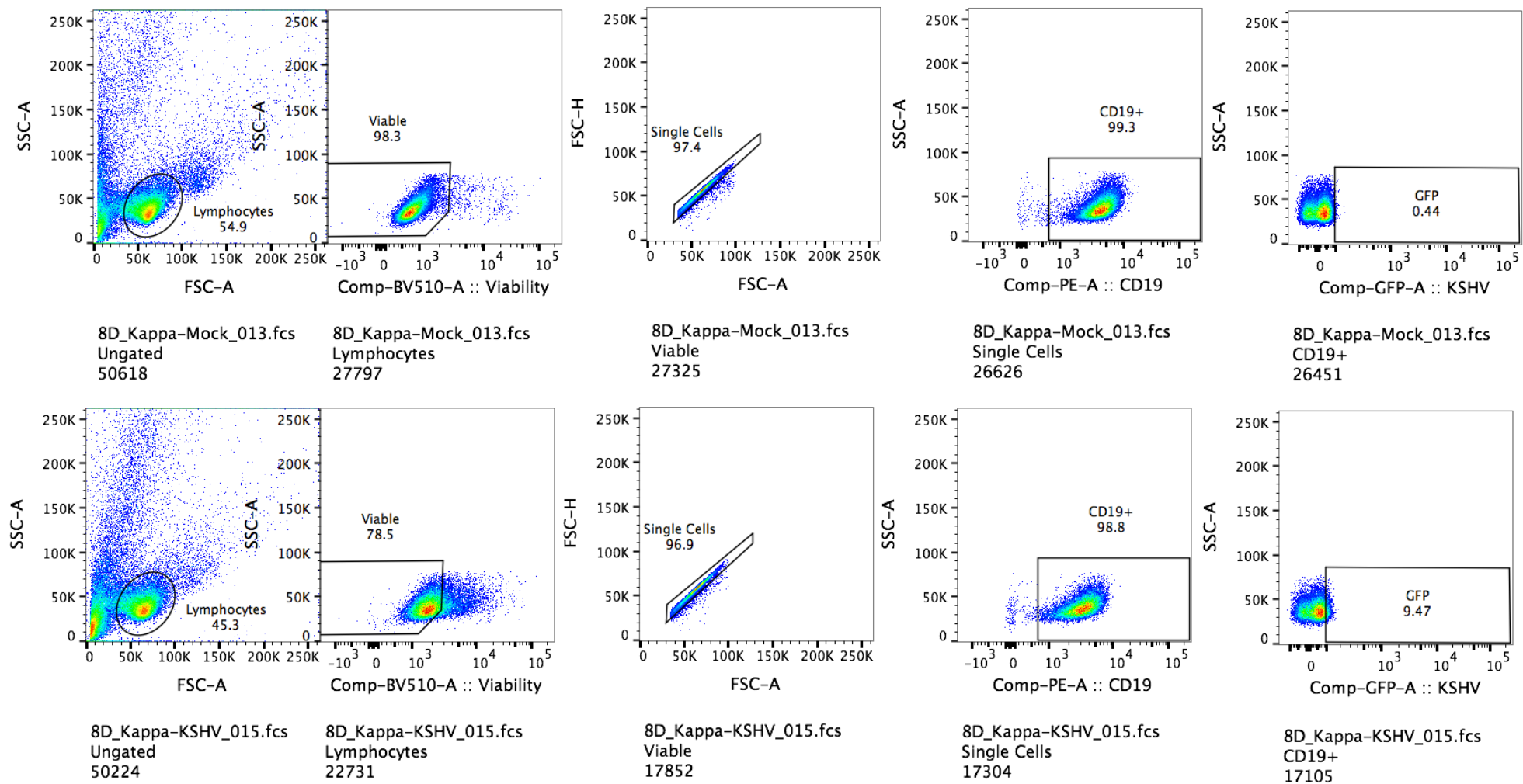

B

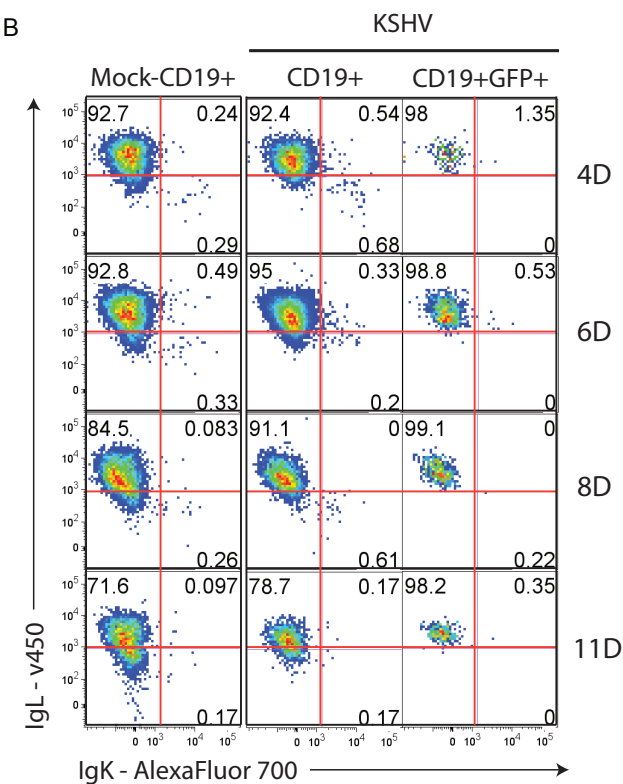

Supplement: S2 Fig — (A) Full gating scheme for flow sorted BCR revision experiments shown in Fig 4 (B) Igλ+ Naive B lymphocytes were sorted, infected with KSHV and analyzed in parallel with Igκ lymphocytes shown in Fig 4. (PDF) [file ppat.1006967.s002.pdf]

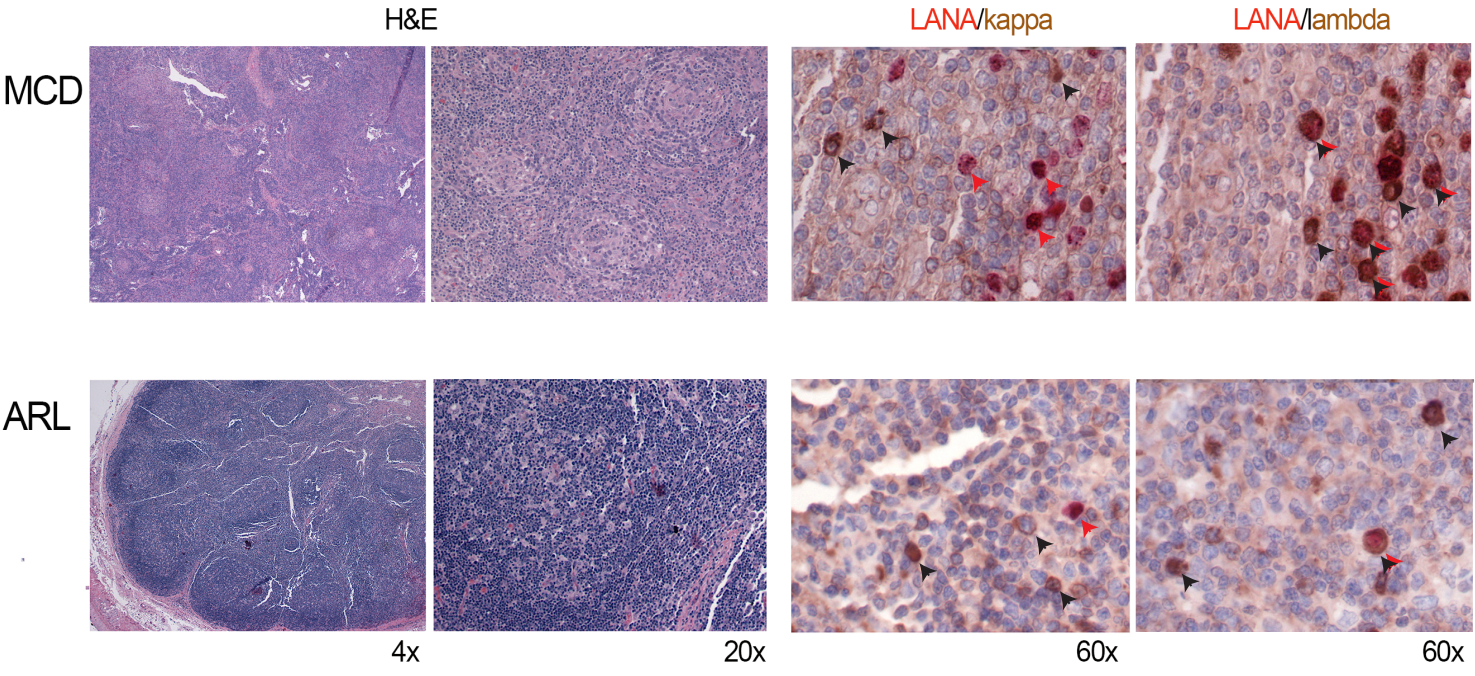

Supplement: S3 Fig — Primary samples with H&E staining at 4x and 20x and corresponding immunohistochemistry for LANA (red) and immunoglobulin light chains (brown) demonstrating that both KSHV-infected lymphocytes (red arrows) in MCD (top) and non-MCD AIDS-related lymphadenopathy (bottom) do not express Igκ (black arrows, left) but are positive for Igλ (black arrows, right). (PDF) [file ppat.1006967.s003.pdf]

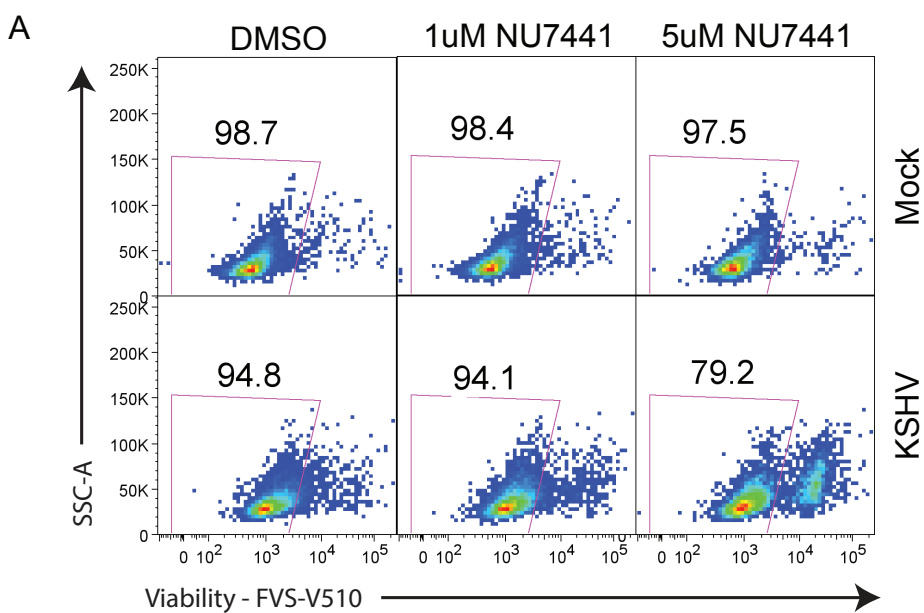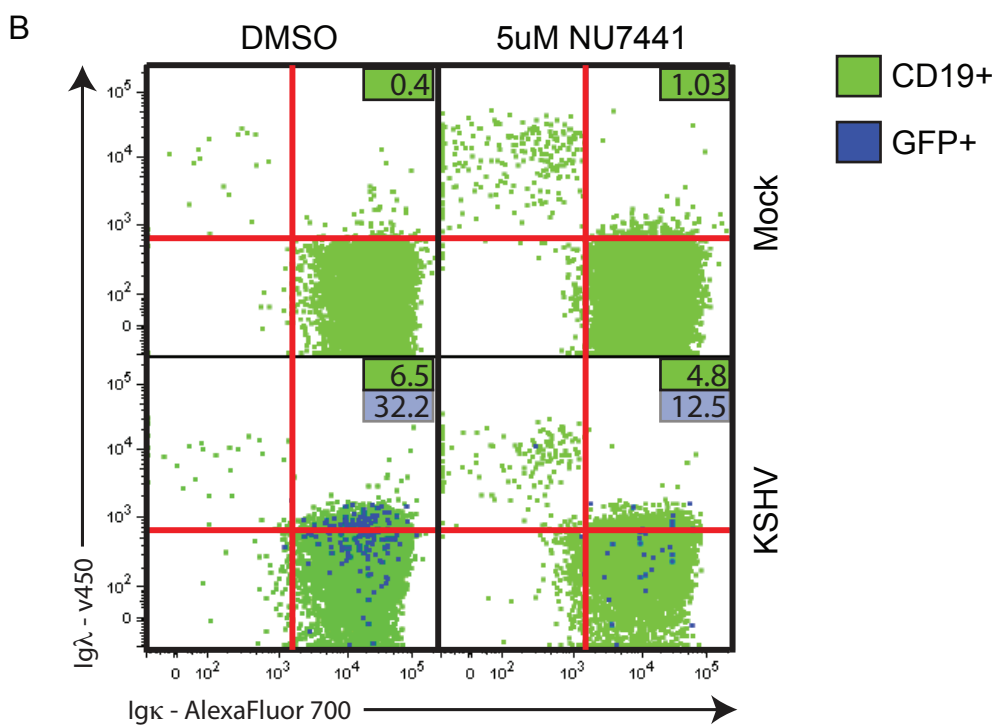

Supplement: S4 Fig — Naive B lymphocytes were flow sorted based on Igκ-expression and pre-treated with DMSO or 5μM NU7441 for 1 hour. Cells were subsequently infected mock-infected or infected with KSHV in the presence of treatments and plated on irradiated CDw32 feeder cells. At 5 days post-infection cells were harvested and analyzed by FACS for (A) cell viability using an exclusion dye and (B) light chain expression. Singlet-gated viable cells were included in the analysis. For (B) light chain expression for total CD19+ (green) and CD19+, GFP+ (blue) in a representative experiment is shown. (PDF) [file ppat.1006967.s004.pdf]
